# Drug-eluting Vein Graft with Acetylsalicylic Acid-Ticagrelor-Unfractionated Heparin Complex Inhibits Early Graft Thrombosis

**DOI:** 10.4274/balkanmedj.galenos.2020.2020.1.128

**Published:** 2020-08-11

**Authors:** Ercan Akşit, Tolga Kurt, Başak Büyük, Ömer Çokkalender

**Affiliations:** 1 Department of Cardiology, Çanakkale Onsekiz Mart University School of Medicine, Çanakkale, Turkey; 2 Department of Cardiovascular Surgery, Çanakkale Onsekiz Mart University School of Medicine, Çanakkale, Turkey; 3 Department of Histology and Embryology, İzmir Demokrasi University School of Medicine, İzmir, Turkey; 4 Clinic of Cardiovascular Surgery, 25 Aralık State Hospital, Gaziantep, Turkey

**Keywords:** Acetyl salicylic acid, heparin, hydrogel, thrombosis, ticagrelor, vein bypass graft

## Abstract

**Background:**

Bypass graft surgery remains to be an important treatment option for left main and multivessel coronary artery disease. Approximately 2% of saphenous vein grafts are lost immediately after the coronary artery bypass graft operations and 12% in the first month due to thrombosis.

**Aims:**

To administer one anticoagulant and two antiplatelet agents in a way that locally affects the vein graft before the bypass operation and to thereby analyse their effects on early graft thrombosis.

**Study Design:**

Animal experimentation.

**Methods:**

Since ticagrelor was used locally for the first time in this study, its efficacy in combination with other drugs (acetylsalicylic acid, acetylsalicylic acid and ticagrelor, and acetylsalicylic acid + ticagrelor + unfractionated heparin) was examined on rats including control (untreated) and sham (pluronic gel) group (n=14 for each group). Before the tunica adventitia layer of the femoral veins was bypassed to the femoral artery, it was coated with the drug-eluting pluronic F-127 gel. The presence or absence of thrombus in the vein graft samples was recorded under light microscopy. In vein graft preparations where thrombus was detected, the thrombus area (μm^2^) was calculated using the Axiovision software. Immunohistochemical staining was performed with the anti-rat von Willebrand factor polyclonal antibody kit.

**Results:**

The number of preparations containing thrombus was significantly lower in the acetylsalicylic acid + ticagrelor + unfractionated heparin group than in the acetylsalicylic acid, control, and sham groups, according to the comparisons made on the 1^st^ and 3^rd^ days (p=0.001 and 0.02, respectively). von Willebrand factor staining was significantly lower in the acetylsalicylic acid + ticagrelor + unfractionated heparin group than in the other groups on the 3^rd^ day (p=0.005).

**Conclusion:**

Locally effective acetylsalicylic acid-ticagrelor-unfractionated heparin complex has been shown to significantly reduce thrombus formation in vein grafts in this experimental model. Local administration of these drugs, which are routinely administered orally just before stent implantations, on the vein graft before the bypass is performed can prevent the loss of vein grafts due to thrombus, thereby reducing the mortality and morbidity of these patients.

Bypass graft surgery remains an important treatment option for left main and multivessel coronary artery disease (CAD) (1). Although the durability of arterial grafts obtained for a single lesion is good, vein grafts are still frequently used for multivessel disease, and 80% of the grafts used in coronary artery bypass graft (CABG) operations are venous (2).

Approximately 2% of saphenous vein grafts (SVGs) are lost immediately after the CABG operation and 12% in the first month due to thrombosis (3). Although guidelines suggest using multiple arteries to bypass veins, real-life data in North America showed that it could only be used in 9% of CABG operations (4). Thus, treatment modalities that will prevent clogging of the saphenous veins, which are still the cornerstones for multivessel bypass surgery, should be developed (5).

In percutaneous coronary interventions, class 1 recommendations to prevent stent thrombosis include one anticoagulant and two antiplatelet agents, and they should be administered to the patient at the first contact with the loading dose to initiate the effect as soon as possible (1). The purpose of this study is to administer one anticoagulant and two antiplatelet agents in a way that locally affects the vein graft before the bypass and to thereby analyse their effects on early graft thrombosis.

## MATERIALS AND METHODS

### Experimental animals

The study was approved by the Institutional Animal Use and Care Committee of Çanakkale Onsekiz Mart University and conducted following the Helsinki Declaration of the World Medical Association recommendations on animal studies (protocol no: 2018/02-01). This study used 70 Wistar albino male rats aged 4-6 months and weighed between 300 and 350 g. Standard rat feed and water were provided throughout the study. The rats were kept in a special steel cage at a temperature of 22°C±2°C in a special room, with a humidity of 50%-55% and a 12-h dark/light cycle.

### Hydrogel and drugs

Pluronic F-127 (Sigma-Aldrich, Steinheim am Albuch, Germany, lot no: BCBW5376), a hydrogel with a controlled release of approximately 3 days, was selected as the local delivery medium. The hydrogel was prepared as described in the literature (6), and the gel preparation instructions in the product leaflet were followed. For this purpose, 2 g of pluronic F-127 was dissolved in 10 mL of dimethyl sulfoxide and heated at 40°C for 20 min to obtain a 20% (w/v) stock solution. Acetylsalicylic acid (ASA) (Kimetsan, Ankara, Turkey, KIM-ASA/01PG/180629) was chosen as the first antiplatelet agent of the study; 100 µg of ASA active agent was dissolved in 1 mL of 20% pluronic-127 gel (pH 7.2) (7). Since no prodrug inhibits adenosine diphosphate (ADP) receptors (8), ticagrelor (Sigma-Aldrich, lot no: 1390403), which can act locally, was chosen as the second antiplatelet agent; 100 µg of ticagrelor active agent was dissolved in 1 mL of 20% pluronic-127 gel (pH 7.2). Since ticagrelor was applied locally for the first time, the dose was calculated using the respective amount of ticagrelor on gram tissue based on systemic ticagrelor administration performed previously in the experimental study by Preusch et al. (9) that used a systemic dose of 270 mg/kg. Our local dose calculation was performed by considering the graft weight (0.37 mg); therefore, the local dose of ticagrelor was calculated to be 100 µg. Unfractionated heparin (UFH) (Polifarma) was chosen as the anticoagulant. The UFH dose that was locally used in a previous study was taken as reference (10). Thus, 100 units/mL of UFH was prepared in 20% pluronic-127 gel (pH 7.2).

### Study groups

Since ticagrelor was used locally for the first time in this study, its efficacy with combinations of other drugs (only ASA, ASA + ticagrelor, and ASA + ticagrelor + UFH) was examined on rats including the control (untreated) and sham (pluronic gel) groups. Since ASA was shown to be locally effective in vein grafts (7), and we wanted to test whether drug combinations with ticagrelor have any superiority against ASA treatment alone, the Wistar albino rats were randomized into five basic groups. The samples were divided according to the thrombus area data obtained from the study of Torsney et al. (7). There was a 40% reduction after comparing the baseline and post-interventional results. Power analysis performed with 80% power and an alpha error of 5% according to those results revealed that groups should be composed of at least 14 subjects.

The different groups are as follows: group I (control, n=14): Only bypass operation was performed, and no local treatment was provided to this group. Group II (n=14): ASA was locally applied to the vein graft on which bypass operation was performed. Group III (n=14): ASA + ticagrelor were locally applied to the vein graft on which bypass operation was performed. Group IV (n=14): ASA + ticagrelor + UFH were locally applied to the vein graft on which the bypass operation was performed. Group V (sham, n=14): Only pluronic gel was locally applied to the vein graft on which bypass operation was performed. In all the groups, the tissue samples were obtained on the 1^st^ (n=7) and 3^rd^ days (n=7).

### Anesthesia protocol

Anesthesia was induced by intraperitoneally administering ketamine hydrochloride (75 mg/kg) and xylazine (5-10 mg/kg) to the rats.

### Bypass graft model

Two experienced cardiovascular surgeons performed the bypass operation. The surgeons initiated the surgical procedure after establishing a vision with a 3.5x magnification surgical loupe. To protect the rats from infection, preoperative cefazolin sodium (0.5 g/kg) was intramuscularly administered. The modified bypass model was performed by interpositioning the femoral vein to the femoral artery of the other leg using the end-to-side technique, as described previously (11). Before the tunica adventitia layer of the femoral veins was bypassed to the femoral artery, it was coated with the drug-eluting pluronic F-127 gel. This hydrogel, which is solid at room temperature because of its chemical properties, begins the controlled release of the drug for 3 days at body temperature (6,7). Vein graft samples were obtained from the rats on the 1^st^ and 3^rd^ days after the procedure. Euthanasia was performed by cervical dislocation.

### Histopathological evaluation

A histologist who was blinded to the experimental procedure performed the histopathological evaluation in the Çanakkale Onsekiz Mart University Histology Department. Vein graft tissue samples were fixed in 10% neutral buffered formalin for 48 h. After fixating, dehydrating, clearing, and paraffin embedding, 5-micron-thick sections were obtained from the paraffin blocks using Rotary Microtome (Leica RM2125 RTS, Germany). Sections were placed on slides so that the endothelial surfaces of the vein grafts were facing up. The samples were evaluated under a light microscope (Zeiss, USA). Perivascular inflammation was graded by routine hematoxylin and eosin staining in tissue sections.

Histopathological grading was performed by quantitative scoring between 0 and 3. Scoring was performed as follows: 0, no findings within the light microscopy field examined; 1, findings in less than 25% of the field; 2, findings in 25%-75% of the field; and 3, findings in more than 75% of the field (12). The presence or absence of thrombus in the vein graft samples was recorded under light microscopy. In vein graft preparations where thrombus was detected, the thrombus area (µm^2^) was calculated using Axiovision software (Zeiss).

### Immunohistochemical staining

Immunohistochemical staining was performed with the anti-rat von Willebrand factor (VWF) polyclonal antibody kit (Bioss, USA, lot no: AD071046) following the manufacturer’s instructions. Immunohistochemical scoring of VWF was performed according to the staining intensity of the preparations (−, negative; +, positive; ++, strongly positive; and +++, very strongly positive) (13).

### Calculation of ADP receptor level in serum

Serum ADP receptor level was examined in the blood samples to check whether ticagrelor had entered the systemic circulation in the current dose. Blood samples were obtained from the tail veins of each animal just before euthanasia. ADP receptor inhibition level was analyzed using the P2YR12-ADP receptor enzyme-linked immunosorbent assay kit (Sunredbio, China, lot no: 201904) following the manufacturer’s instructions.

### Statistical analysis

In summarizing the data obtained from the study, descriptive statistics for continuous variables were presented as median and interquartile range according to data distribution. Categorical variables were presented as numbers and percentages. The normality of numerical variables was evaluated with a one-sample Kolmogorov-Smirnov test. The Fisher-Freeman-Halton test compared perivascular inflammation, VWF staining, and thrombus presence; whereas the Kruskal-Wallis test compared thrombus area and ADP receptor level between the groups. In the presence of significant differences with more than two group comparisons, post-hoc pairwise comparisons were performed via the Bonferroni correction method. Jamovi (2018, Version 1.0.5) and JASP Team (2018, Version 0.10.2) software were used for statistical analyses, and a p-value of <0.05 was considered statistically significant.

## RESULTS

### Histopathologic findings

Thrombus was observed in four preparations (57%) each on days 1 and 3 in the ASA + ticagrelor group and in two (29%) and four preparations (57%) in days 1 and 3, respectively, in the ASA + ticagrelor + UFH group. All preparations in the other groups had thrombi on days 1 and 3 (p=0.001 and 0.02, respectively). Considering the calculated thrombus areas for preparations that exhibited thrombus, the lowest median values were also observed in the ASA + ticagrelor [day 1 (540.3 µm^2^) and day 3 (3522.7 µm^2^)] and the ASA + ticagrelor + UFH [day 1 (0 µm^2^) and day 3 (1092 µm^2^)] groups, but there was no statistically significant difference between any of the groups (p=0.086 and 0.174 for days 1 and 3, respectively).

**Figure 1:**
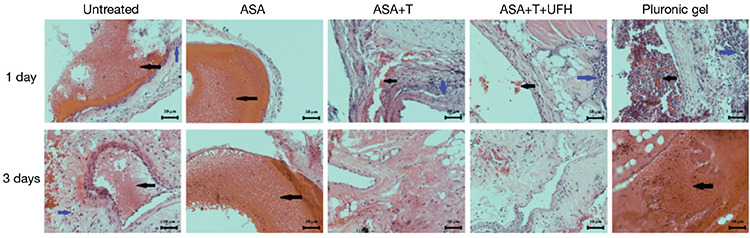
Hematoxylin-eosin-stained microscopic images of the study groups on the 1^st^ and 3^rd^ days. After routine histopathological evaluation, perivascular inflammation is indicated by blue arrows, and thrombus formation is indicated by black arrows. It was observed that perivascular inflammation and thrombus formation were significantly reduced with the addition of ticagrelor to acetylsalicylic acid (ASA). From a general point of view, the lowest rate of perivascular inflammation and thrombus formation was observed in the group that contained ASA + ticagrelor + unfractionated heparin (magnification 200x).

In terms of perivascular inflammation, two preparations (29%) in the ASA + ticagrelor + UFH group did not exhibit any inflammation on day 1, whereas all preparations had inflammation in the other groups. Moreover, none of the preparations exhibited intense inflammation (grade 3) in the ASA + ticagrelor + UFH and ASA + ticagrelor groups (p=0.004). One preparation (14%) did not exhibit any inflammation on day 3 in the ASA + ticagrelor + UFH group. Although none of the preparations exhibited intense inflammation in the ASA + ticagrelor group, it was observed in only one preparation (14%) in the ASA + ticagrelor + UFH group (p=0.408; Figure 1 and Table 1).

### Immunohistochemical findings

**Figure 2:**
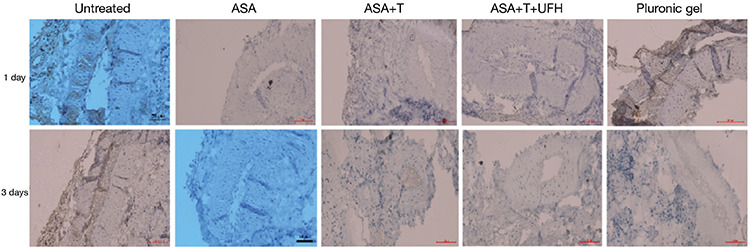
Von Willebrand factor (VWF) immunohistochemical staining of the study groups on the 1^st^ and 3^rd^ days. It was observed that VWF staining was lower in the locally acting drug group compared with that in the untreated and pluronic gel groups. From a general point of view, the lowest staining was observed with preparations containing acetylsalicylic acid + ticagrelor + unfractionated heparin (magnification 200x).

No VWF staining was observed in one preparation (14%) in the ASA group, two (29%) in the ASA + ticagrelor group, and five (71%) in the ASA + ticagrelor + UFH group on day 1, whereas all preparations exhibited VWF staining in the other groups. In addition, none of the preparations in the ASA, ASA + ticagrelor, and ASA + ticagrelor + UFH groups exhibited very strong (+++) staining. None of the preparations were strongly stained (++) in the ASA + ticagrelor + UFH group, and all preparations exhibited strong staining in the other groups (p=0.004). There was no VWF staining in one preparation (14%) in the ASA group, three (43%) in the ASA + ticagrelor group, and six (86%) in the ASA + ticagrelor + UFH group on day 3, whereas all preparations exhibited VWF staining in the other groups. None of the preparations in the ASA, ASA + ticagrelor, and ASA + ticagrelor + UFH groups exhibited very strong (+++) staining, and none of the preparations in the ASA + ticagrelor and ASA + ticagrelor + UFH groups exhibited strong (++) staining (p=0.005; Table 1 and Figure 2).

### ADP receptor levels

Considering the serum ADP receptor levels, the lowest median values were observed in the ASA + ticagrelor [day 1 (88.2 pg/mL) and day 3 (83.2 pg/mL)] and ASA + ticagrelor + UFH [day 1 (82.7 pg/mL) and day 3 (86.1 pg/mL)] groups, but there was no statistically significant difference between any of the groups (p=0.331 and 0.793 for days 1 and 3, respectively).

## DISCUSSION

The present study showed that the locally effective ASA-Ticagrelor-UFH complex reduces thrombus formation in vein grafts and results in less VWF staining compared with other treatment modalities. Studies have shown that there is intense platelet, neutrophil migration, and fibrin accumulation into the vein wall in the first hours after anastomosis of the vein graft to the coronary artery, leading to intimal hyperplasia after treatment (14,15). Aiming to prevent this mechanism from the very beginning appears to slow down the subsequent processes (7). The absence of antiplatelet and anticoagulant agents during the first hours after CABG may be associated with thrombosis development (14). In a recent study, it has been shown that ASA, which is systemically administered, causes an increase in chest tube drainage and thus, surgical re-exploration (16). Since systemic administration of drugs causes adverse events, researchers tried local drug administration to prevent SVG disease. In their study, Torsney et al. (7) showed that local ASA application reduced thrombus formation in vein grafts. In this experimental model, they used hydrogels with up to 3 days of controlled release that is similar to our study, and they showed that the local effect continued for approximately 1 month and that local ASA also reduced neointimal formation (7). In the present study, we found that the samples with the least thrombus formation were those containing the ASA-Ticagrelor-UFH complex.

Ticagrelor is not a prodrug, and it is reversibly bound to the ADP receptor, and its efficacy is not affected by genetic polymorphism. Ticagrelor is the fastest agent that inhibits platelet aggregation. Its adenosine-dependent pleotropic effect is its most interesting feature (8). Grzesk et al. (17) found that orally administered ticagrelor prevented ADP-dependent vascular smooth muscle cell contraction, unlike clopidogrel and prasugrel. Ticagrelor is also one of the ADP receptor blockers that reduce cardiovascular mortality without increasing bleeding complications after a bypass operation (18). In the present study, no adverse effects of the drugs were detected in the subjects, and no difference was noted between the groups in terms of serum ADP receptor levels at the local ticagrelor dose used in the study. Furthermore, we found that there were fewer thrombi in the ASA and ticagrelor combination treatment group. The superiority of the ASA and ticagrelor combination may be explained by pleotropic actions and the fact that ticagrelor had direct effects (because it is not a prodrug) on tissues. Considering that the most frequently occurring pathogens in these graft infections are *Staphylococcus aureus* and *Staphylococcus epidermidis*, the antibacterial effect of local ticagrelor in addition to reducing thrombus formation may contribute to the reduction of graft infections as well (19,20).

Local use of UFH, the anticoagulant agent chosen for the present study, has been previously found to prevent arterial thrombosis. Andresen et al. (10) showed that local heparin reduced arterial thrombosis in microvascular surgery more than IV heparin and that no significant difference was noted between the control groups in terms of serum activated partial thromboplastin time levels. VWF is an important molecule that plays a significant role in thrombus formation, is used as an endothelial activation marker, and predicts mortality in patients with cardiovascular events and especially previously known CAD (21). Furthermore, some studies have found that VWF levels are a marker of damage to the vein during harvest (22). In a study by Meyer et al. (23), strong immunohistochemical staining was determined in the endothelium of almost all SVGs for factor VIII. In the present study, VWF staining was at significantly lower levels in the ASA + Ticagrelor + UFH group than in the other groups; furthermore, there was a continuous decrease in the staining levels from the untreated group to the three-drug combination group.

Wu et al. (24) showed that resolvin D1 loaded on a hydrogel reduced neointimal hyperplasia in vein grafts. Although there are many experimental studies, the only human trial has been conducted with edifoligide; however, no significant difference was noted between the two groups in terms of SVG loss (25). Drug-eluting stents are loaded with agents that reduce intimal hyperplasia (26); however, since reducing thrombus burden is also known to reduce future intimal hyperplasia (7), it appears more reasonable to first develop local treatment modalities to reduce thrombus formation to prevent SVG disease. The difference of our study from these studies is that it targets thrombus formation, the first mechanism of graft loss, and includes a two antiplatelet, one anticoagulant regimen, which is similar to the guidelines aimed at preventing stent thrombosis.

This method is cost-effective because of the very low amount of hydrogel used and very low drug doses administered. Moreover, hydrogels have been used for controlled drug delivery in the pharmaceutical industry for many years (6). This method is also practical, as we described in the Method section. Drugs can be prepared in hydrogel beforehand, and the adventitia layer of the vein graft can be coated with a hydrogel containing drugs before the bypass.

This study has some limitations. Since a hydrogel with controlled release for 3 days was chosen as the local distribution tool in the present study, tissue and blood samples were obtained from the subjects only on days 1 and 3. Since the effect of drug combinations was investigated, subjects were randomized into too many groups. The effect of drugs on thrombus development could not be investigated in consecutive weeks for up to 1 month because of ethical reasons since the number of subjects would increase too much. We could not examine histopathologic markers other than VWF staining because of funding limitations.

In conclusion, a locally effective ASA-Ticagrelor-UFH complex has been shown to significantly reduce thrombus formation in vein grafts in this experimental model. Local administration of these drugs, which are routinely administered orally just before stent implantations, on the vein graft before the bypass can prevent the loss of vein grafts due to thrombus, thereby reducing the mortality and morbidity of these patients.

## Figures and Tables

**Table 1:**
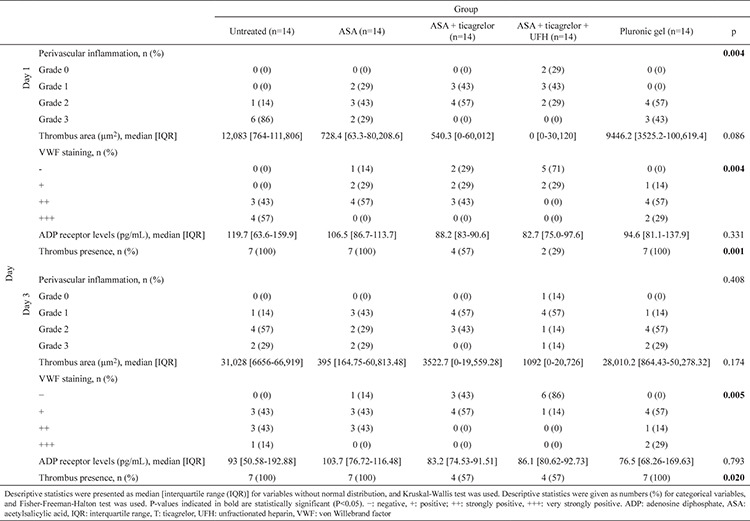
Comparison of perivascular inflammation, thrombus area, von Willebrand factor staining, adenosine diphosphate receptor inhibition level, and thrombus presence between the groups
